# Melanopsin Regulates Both Sleep-Promoting and Arousal-Promoting Responses to Light

**DOI:** 10.1371/journal.pbio.1002482

**Published:** 2016-06-08

**Authors:** Violetta Pilorz, Shu K. E. Tam, Steven Hughes, Carina A. Pothecary, Aarti Jagannath, Mark W. Hankins, David M. Bannerman, Stafford L. Lightman, Vladyslav V. Vyazovskiy, Patrick M. Nolan, Russell G. Foster, Stuart N. Peirson

**Affiliations:** 1 Sleep and Circadian Neuroscience Institute (SCNi), Nuffield Department of Clinical Neurosciences, Oxford Molecular Pathology Institute, Dunn School of Pathology, University of Oxford, Oxford, United Kingdom; 2 Department of Experimental Psychology, University of Oxford, Oxford, United Kingdom; 3 Henry Wellcome Laboratories for Integrative Neuroscience and Endocrinology, University of Bristol, Bristol, United Kingdom; 4 Department of Physiology, Anatomy and Genetics, University of Oxford, Oxford, United Kingdom; 5 MRC Harwell, Harwell Science and Innovation Campus, Oxfordshire, United Kingdom; Strasbourg University and CNRS UPR 3212, FRANCE

## Abstract

Light plays a critical role in the regulation of numerous aspects of physiology and behaviour, including the entrainment of circadian rhythms and the regulation of sleep. These responses involve melanopsin (OPN4)-expressing photosensitive retinal ganglion cells (pRGCs) in addition to rods and cones. Nocturnal light exposure in rodents has been shown to result in rapid sleep induction, in which melanopsin plays a key role. However, studies have also shown that light exposure can result in elevated corticosterone, a response that is not compatible with sleep. To investigate these contradictory findings and to dissect the relative contribution of pRGCs and rods/cones, we assessed the effects of light of different wavelengths on behaviourally defined sleep. Here, we show that blue light (470 nm) causes behavioural arousal, elevating corticosterone and delaying sleep onset. By contrast, green light (530 nm) produces rapid sleep induction. Compared to wildtype mice, these responses are altered in melanopsin-deficient mice (*Opn4*^*-/-*^), resulting in enhanced sleep in response to blue light but delayed sleep induction in response to green or white light. We go on to show that blue light evokes higher *Fos* induction in the SCN compared to the sleep-promoting ventrolateral preoptic area (VLPO), whereas green light produced greater responses in the VLPO. Collectively, our data demonstrates that nocturnal light exposure can have either an arousal- or sleep-promoting effect, and that these responses are melanopsin-mediated via different neural pathways with different spectral sensitivities. These findings raise important questions relating to how artificial light may alter behaviour in both the work and domestic setting.

## Abstract 2

Light can produce either sleep or arousal in mice. This study reveals that these opposing effects depend upon the wavelength of light and appear to involve separate pathways, both modulated by the photopigment melanopsin.

## Author Summary

Light exerts profound effects on our physiology and behaviour, setting our biological clocks to the correct time and regulating when we are asleep and we are awake. The photoreceptors mediating these responses include the rods and cones involved in vision, as well as a subset of photosensitive retinal ganglion cells (pRGCs) expressing the blue light-sensitive photopigment melanopsin. Previous studies have shown that mice lacking melanopsin show impaired sleep in response to light. However, other studies have shown that light increases glucocorticoid release—a response typically associated with stress. To address these contradictory findings, we studied the responses of mice to light of different colours. We found that blue light was aversive, delaying sleep onset and increasing glucocorticoid levels. By contrast, green light led to rapid sleep onset. These different behavioural effects appear to be driven by different neural pathways. Surprisingly, both responses were impaired in mice lacking melanopsin. These data show that light can promote either sleep or arousal. Moreover, they provide the first evidence that melanopsin directly mediates the effects of light on glucocorticoids. This work shows the extent to which light affects our physiology and has important implications for the design and use of artificial light sources.

## Introduction

In addition to its familiar visual function the mammalian retina mediates a broad range of non-image forming responses to light, including the entrainment of circadian rhythms [1], regulation of pineal melatonin synthesis [2], pupillary light constriction [3] and the regulation of sleep [4–6]. Research on these responses led to the identification of a novel retinal photoreceptor, consisting of a subset of photosensitive retinal ganglion cells (pRGCs) expressing the photopigment melanopsin (OPN4) [7,8]. These melanopsin pRGCs project to a wide range of brain targets, which are thought to mediate the effects of light on different aspects of physiology and behaviour. For example, entrainment of circadian rhythms is mediated by projections to the suprachiasmatic nuclei (SCN) [7–9], whereas sleep induction is thought to involve the ventrolateral preoptic area (VLPO) [4–6].

Non–image-forming responses to light persist in the absence of rods and cones [1,2], demonstrating a critical role of melanopsin pRGCs in these processes. However, loss of melanopsin does not abolish these responses, indicating that rods and cones can at least partially compensate for the absence of pRGC photosensitivity [7,10,11]. By contrast, loss of the pRGCs themselves produces a dramatic loss of non–image-forming responses, suggesting that these cells provide the principal conduit for the light input from rods and cones [12]. As such, whilst melanopsin pRGCs play a critical role in mediating non–image-forming responses to light, it is clear that these cells also receive inputs from rods and cones, which contribute to these processes [13–17]. Moreover, recent studies have shown that visual responses may also be modulated by irradiance information detected by melanopsin, for example, in light adaptation [18].

Surprisingly, given the role of melanopsin in the regulation of light-induced sleep, nocturnal light exposure in rodents has also been shown to result in a rise in plasma corticosterone [19,20], an arousal response that is physiologically incompatible with sleep induction [21]. To date, no studies have addressed the relationship between the sleep- and arousal-promoting effects of acute light exposure in rodents, nor investigated the role of melanopsin in these responses.

Here, we investigate the effects of different wavelengths of light on sleep induction to determine if there is a differential effect of wavelength on sleep versus arousal. In view of previous findings, we were surprised to find that blue light mediates behavioural light aversion and elevated corticosterone via melanopsin pRGCs, whilst green light results in rapid sleep induction. Significantly, sleep induction in response to blue light is actually enhanced in *Opn4*^*-/-*^ animals. Collectively, our data demonstrate that different wavelengths of light exert different effects on sleep and arousal, and these opposing responses are mediated by different photoreceptor pathways. Our data suggest that the role of melanopsin in the regulation of sleep is much more complex than originally envisaged.

## Results

### The Effects of Light on Sleep Induction Are Wavelength-Dependent

Previous studies have shown that sleep induction in melanopsin knockout mice (*Opn4*^*-/-*^) in response to nocturnal white light stimuli is attenuated [4–6]. These data suggest a critical role for melanopsin in the regulation of sleep. To investigate this further, we studied behaviourally-defined sleep induction in response to different wavelengths in C57BL/6 mice. To prevent confounds from animals already being asleep, studies were performed at ZT14 when sleep pressure is low and animals show highest activity levels. In mice, the maximum absorption of rod opsin peaks at 498 nm [22] with M-cone opsin at 508 nm [23], S-cone opsin around 360 nm [24], and melanopsin around 480 nm [3]. We studied sleep onset in response to a 1 h isoquantal light exposure at ZT14 in wildtype C57BL/6 mice. We used three different wavelength light stimuli—violet (405 nm), blue (470 nm), and green (530 nm) (S1A Fig) to produce differential activation of UVS cones, melanopsin, and rods/MWS cones (S1B Fig). Given existing data on the role of melanopsin in sleep regulation, we predicted that blue light would result in the most rapid sleep induction, as this wavelength most closely corresponds to the peak sensitivity of melanopsin (~480 nm).

**Fig 1:**
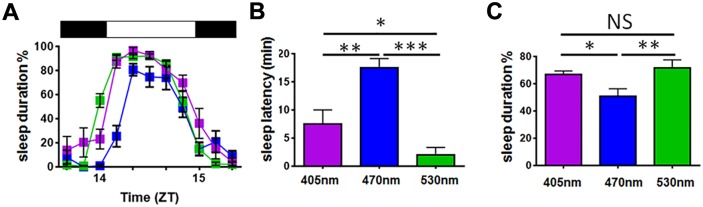
Wavelength-dependent effects on light on sleep induction and sleep duration. (**A**) Mice exposed to different wavelengths (405 nm violet, 470 nm blue, or 530 nm green) for 1 hr at ZT14 exhibited differences in sleep onset and sleep duration. (**B**) Sleep induction is delayed in response to blue light exposure. One-way ANOVA for wavelength, F_(2.23)_ = 18.791, *p* ≤ 0.001. Posthoc Tukey violet versus blue *p* = 0.003, violet versus green *p* = 0.041, blue versus green *p* ≤ 0.001. (**C**) Total sleep duration during the 1 h light pulse is reduced in response to blue light. Data plotted as mean percentage ± SEM (*n* = 8–10/group). Horizontal black-white-black bar illustrates the light pulse condition from ZT14 until ZT15. One-way ANOVA for wavelength, F_(2.23)_ = 4.391, *p* = 0.024. Posthoc Tukey violet versus blue *p* = 0.046, violet versus green *p* = 0.517, blue versus green *p* = 0.008. Statistical differences indicated by *** *p* ≤ 0.001, ** *p* ≤ 0.01, * *p* ≤ 0.05, NS = not significant. The data used to make this figure can be found in S1 Data.

All three wavelengths of light resulted in sleep induction at ZT14, but with different latencies (Fig 1A and 1B). Green light produced a very rapid sleep onset, which was observed almost immediately after light onset (2 ± 1.33 min). By contrast, both violet and blue light showed delayed sleep onset (Fig 1B). Contrary to our prediction, sleep onset in response to blue light was significantly delayed (17. 5 ± 1.64 min) compared to either violet (7.5 ± 2.5 min) or green light exposure (S1, S2, S3 and S4 Videos). This delayed sleep induction under blue light resulted in a reduction of total sleep duration during the 1 h light pulse compared to either violet or green stimuli (Fig 1C). To confirm that this effect was consistent at different times, sleep induction in response to blue and green light was also studied at ZT22. A comparable delay in sleep induction in response to blue light was also observed at this later time (S2 Fig), although differences were not as dramatic. This is most likely due to preceding differences in sleep/wake behaviour during the nocturnal active phase.

### Role of Melanopsin in Sleep Induction

**Fig 2:**
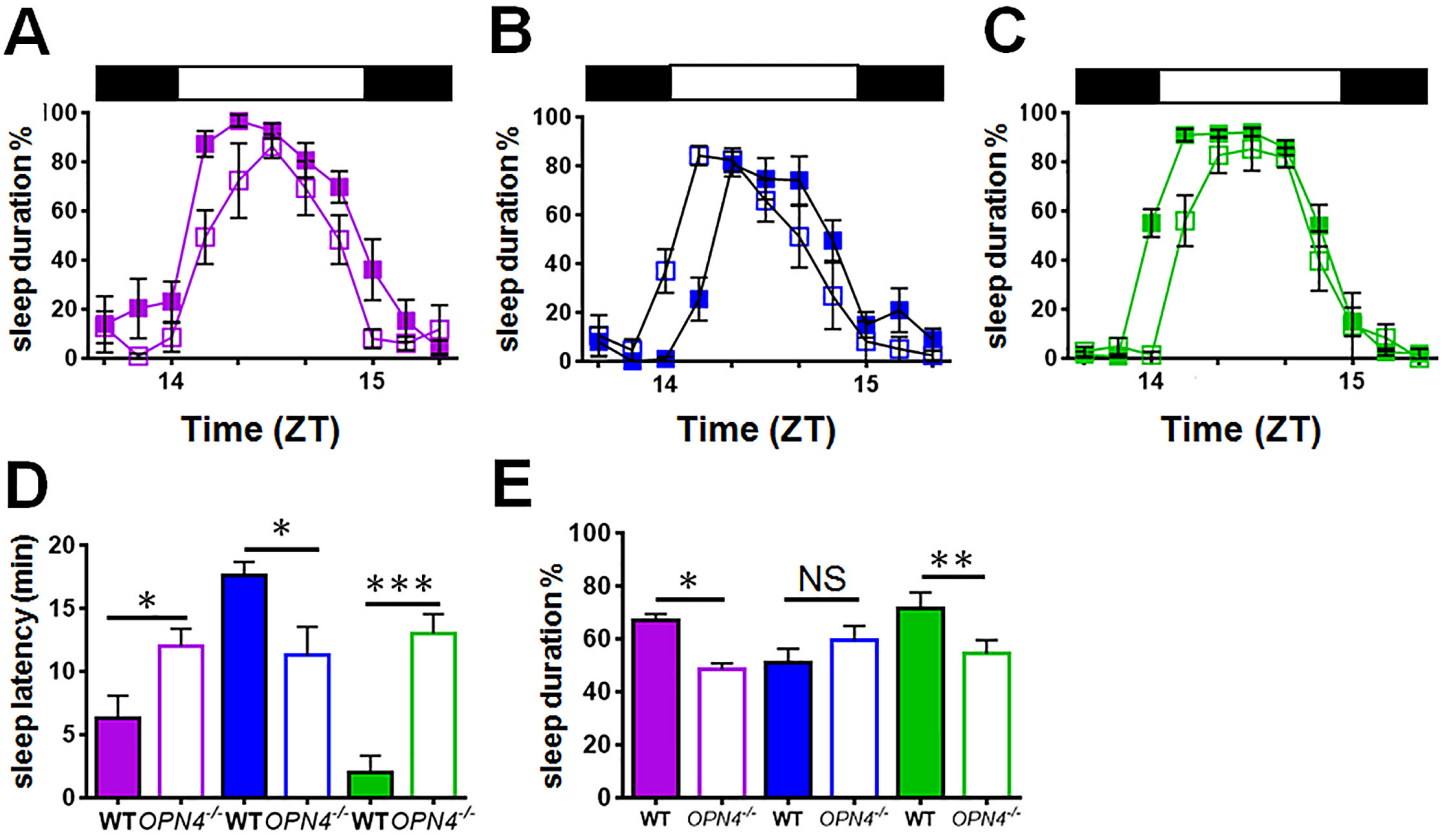
Wavelength-dependent effects of light on sleep are abolished in melanopsin-deficient (*Opn4*^*-/-*^) mice. (**A–C**) Sleep induction in response to violet (**A**), blue (**B**) and green (**C**) light. Sleep was studied in *Opn4*^*-/-*^ mice (open symbols, *n* = 6–10/group) and compared with wildtype mice (WT, solid symbols). (**D**) In comparison with wildtype mice, *Opn4*^*-/-*^ mice show delayed sleep onset in response to green and violet light but advanced sleep onset in response to blue light. Two-way ANOVA for wavelength and genotype, wavelength x genotype interaction F_(2,43)_ = 12.143, *p* ≤ 0.001. Posthoc Tukey wildtype versus *Opn4*^*-/-*^ violet *p* = 0.011, blue *p* = 0.013, green *p* ≤ 0.001. Comparison of *Opn4*^*-/-*^ responses in sleep induction as well as duration show no statistical differences due to wavelength. (**E**) However, in comparison to wildtype mice *Opn4*^*-/-*^ mice exposed to green and violet light show reduced sleep duration. Two-way ANOVA for wavelength and genotype, wavelength x genotype interaction F_(2.44)_ = 5.142, *p* = 0.010, posthoc Tukey wildtype versus *Opn4*^*-/-*^ violet *p* = 0.017, blue *p* = 0.259, green *p* = 0.003. Despite the difference in sleep onset under blue light in *Opn4*^*-/-*^ and wildtype mice, there was no difference in sleep duration. Solid horizontal bars illustrate light pulse duration from ZT14 until ZT15. Data plotted as mean ± SEM. Significant differences indicated by *** *p* ≤ 0.001, ** *p* ≤ 0.01, * *p* ≤ 0.05, NS = not significant. The data used to make this figure can be found in S2 Data.

To investigate whether the different effects of blue light on sleep and light aversion are mediated via melanopsin, we then performed the same experiments in melanopsin knockout (*Opn4*^*-/-*^) mice, on a congenic C57BL/6 background, using violet, blue and green light (Fig 2A–2C). When compared with wildtype responses, sleep onset in *Opn4*^*-/-*^ mice was significantly advanced under blue light (Fig 2D), whereas under violet and green light it was significantly delayed. Sleep duration was reduced in response to violet and green light in *Opn4*^*-/-*^ mice, but unaffected in response to blue light (Fig 2E). Consistent with previous findings, delayed sleep induction and total sleep duration in response to white light were observed—comparable to the green light condition (S3A–S3C Fig). Overall, when responses to different wavelengths were compared in *Opn4*^*-/-*^ mice, no significant differences were evident (S3D–S3F Fig). These results show that melanopsin is necessary for the acute wavelength-dependent effects of light on sleep.

### The Effects of Light on Behavioural Light Aversion Are Wavelength-Dependent

**Fig 3:**
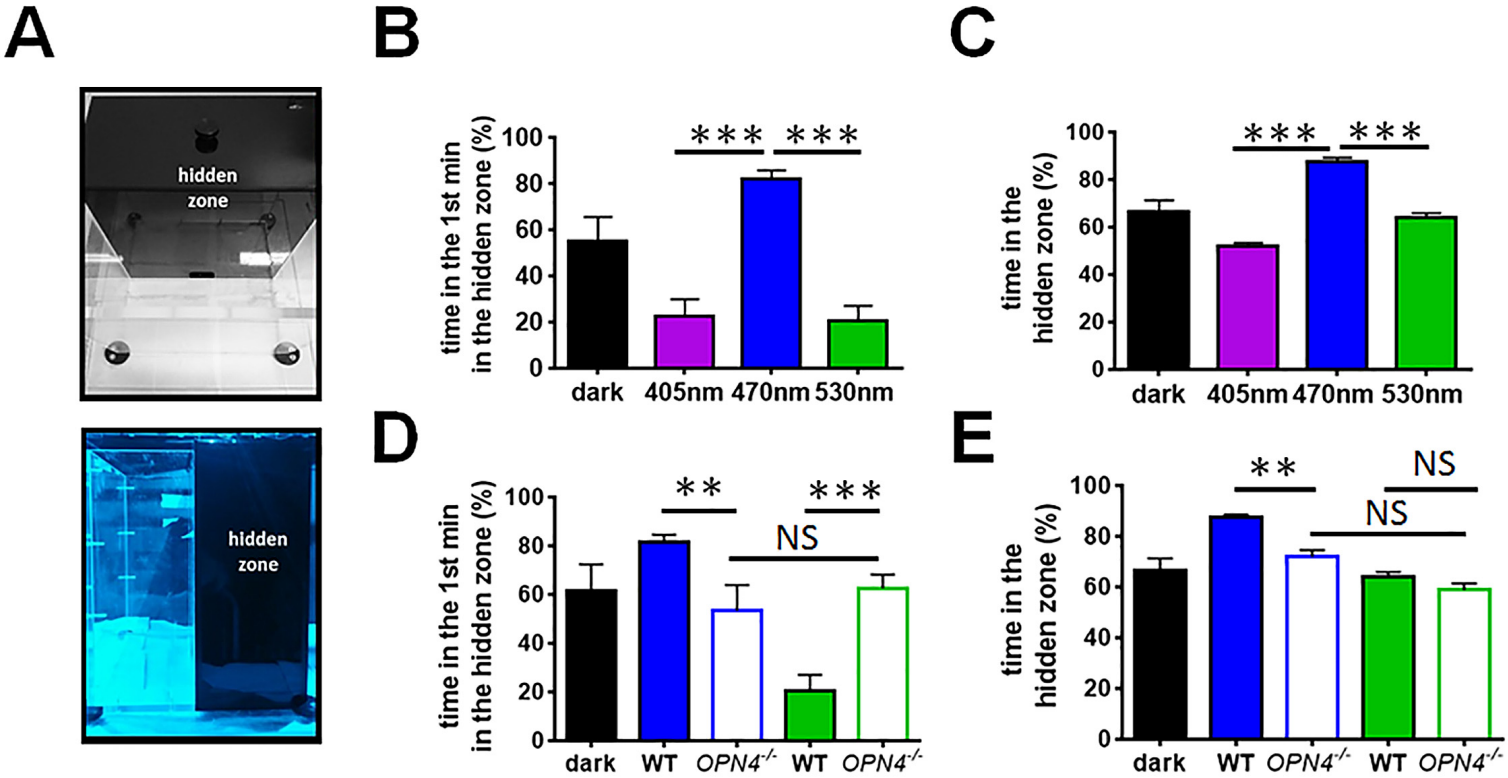
Wavelength-dependent effects on behavioural light aversion. (**A**) Mice entrained to an LD12:12 cycle were exposed to 405 nm (violet), 470 nm (blue), and 530 nm (green) light at ZT14 in a light/dark box for 10 min. (**B**) In the first 1 min of the test, mice spent significantly more time in the hidden zone under blue light compared to violet, green, or control (dark) conditions. One-way repeated measures ANOVA for light condition, F_(3.25)_ = 20.696, *p* ≤ 0.001. Post hoc Tukey dark versus violet *p* = 0.006, dark versus blue *p* = 0.021, dark versus green *p* ≤ 0.001, blue versus violet *p* ≤ 0.001, blue versus green *p* ≤ 0.001. (**C**) In the following 9 min, mice exposed to blue light continued to spend more time in the hidden zone compared to violet or green light. One-way repeated measures ANOVA light condition over time 2–-10 mins, F_(3,25)_ = 11.721, *p* ≤ 0.001. Post hoc Tukey dark versus violet *p* = 0.635, dark versus blue *p* = 0.021, dark versus green *p* = 0.635, violet versus blue *p* ≤ 0.001, blue versus green *p* ≤ 0.001. (**D**) *Opn4*^*-/-*^ mice (open bars) showed different responses to both blue and green light. In the first minute of the test, in comparison with wildtype mice, *Opn4*^*-/-*^ mice spent less time in the hidden zone in response to blue light. However, *Opn4*^*-/-*^ animals spent more time in the hidden zone compared with wildtype under green light. Two-way repeated measures ANOVA for light condition and genotype, light condition x genotype interaction F_(1,26)_ = 20.585, *p* ≤ 0.001. Post hoc Tukey wildtype versus *Opn4*^*-/-*^ blue *p* = 0.010, green *p* = 0.001. (**E**) Whilst the attenuated response to blue light in *Opn4*^*-/-*^ mice persisted over the remaining time course of the test (one-way repeated measures ANOVA for effect of genotype over time, F_(1.13)_ = 14.376, *p* = 0.002), no difference was observed between wildtype and *Opn4*^*-/-*^ mice under green light (one-way repeated measures ANOVA for effect of genotype over time, F_(1.13)_ = 0.26, *p* = 0.617). *Opn4*^*-/-*^ mice showed no statistical difference in responses to wavelength in the first minute or whole time of the test (one-way repeated measures ANOVA for effect time over wavelength F_(9,81)_ = 0.976 *p* = 0.466). Histograms reflect mean percentage ± SEM of time spent in the dark box of the light dark box during the 10 min trial (*n* = 6–-10/group wildtype, *n* = 4–-10 *Opn4*^*-/-*^). Significant differences indicated by *** *p* ≤ 0.001, ** *p* ≤ 0.01, * *p* ≤ 0.05, NS = not significant. The data used to make this figure can be found in S3 Data.

To determine why sleep onset was delayed in response to blue light, we studied behavioural light aversion to investigate if different wavelengths of light were associated with increased anxiety. Behavioural light aversion was measured using the light/dark box at ZT14, comparing the time spent in the hidden (dark) zone of the apparatus when the transparent zone of the box was illuminated with different wavelengths (Fig 3A). Data were analysed based upon the initial response to the test arena (first entry to dark zone and time in dark zone during the first minute of the trial) and over the whole course of the trial (time in the dark zone from 2–10 minutes). A control condition in which both sides of the apparatus were unlit (dark) was also used.

The latency from placing the mouse into the box until the first entry to the dark zone was significantly shorter under blue light (10.98 s ± 1.68) compared to green light (46.52 s ± 4.99), violet (46.47 s ± 4.42), and control (22.95 s ± 6.45) conditions (S4 Fig). During the first minute of testing, mice under blue light spent more time in the hidden zone than under other conditions (Fig 3B). By contrast, during the first minute of testing, mice under green or violet light remained in the illuminated area significantly longer than either the blue light or control group, suggesting that their exploration of this novel environment was not inhibited by light. Over the whole trial, mice spent significantly more time (87.5 ± 2.8%) in the hidden zone under blue light compared to either green or violet light, as well as compared to control conditions (Fig 3C). Under violet or green light, mice spent slightly more than 50% of their time in the dark zone (green 64.15 ± 1.76%, violet 51.91 ± 1.59%) and did not differ from that of control condition where the apparatus was unlit (Fig 3C; control 66.54 ± 2.68%). Overall, these data demonstrate that mice find blue light aversive, both immediately within the first minute and over the 10 min timecourse of the experiment. This aversive effect of light may relate to the delay in sleep onset observed under blue light. By contrast, violet or green light does not evoke an aversive response, and over the first minute of the trial may result in novelty-evoked exploratory behaviour. In the home cage setting, this may in turn relate to the rapid sleep induction in response to green light.

### Role of Melanopsin in Behavioural Light Aversion

We subsequently compared behavioural light aversion in *Opn4*^*-/-*^ mice under blue and green light conditions to determine if melanopsin plays a role in these responses. As we previously found no difference in light aversion between violet and green light (Fig 3B and 3C), violet light was not studied. We found that in comparison to wildtype controls, *Opn4*^*-/-*^ mice showed reduced light aversion to blue light over the whole trial and no difference to green light except in the first minute of the test (Fig 3D and 3E). In the first minute of light exposure (Fig 3D), in comparison to wildtype animals, *Opn4*^*-/-*^ mice spent less time in the hidden zone under blue light, but more time in the hidden zone under green light. Rather than an increase in anxiety, this may simply reflect no preference for either zone (as this was not significantly different to chance), unlike the increased preference for the lit zone seen in wildtype animals. Over the remaining test period (Fig 3E), the reduced aversion to blue light in *Opn4*^*-/-*^ mice was found to persist. However, the difference between wildtype and *Opn4*^*-/-*^ mice in response to green light was not sustained over this period. When light aversion responses to blue and green light were compared in *Opn4*^*-/-*^ mice, no effect of wavelength was observed (Fig 3D and 3E). These data are consistent with the effects of light on sleep in *Opn4*^*-/-*^ mice. The reduced aversive response to blue light in melanopsin-deficient animals is consistent with an advanced sleep onset, whereas the enhanced aversive response to green light at the beginning of the stimulus is consistent with delayed sleep onset.

### The Effects of Light on Gene Expression Are Wavelength-Dependent

**Fig 4:**
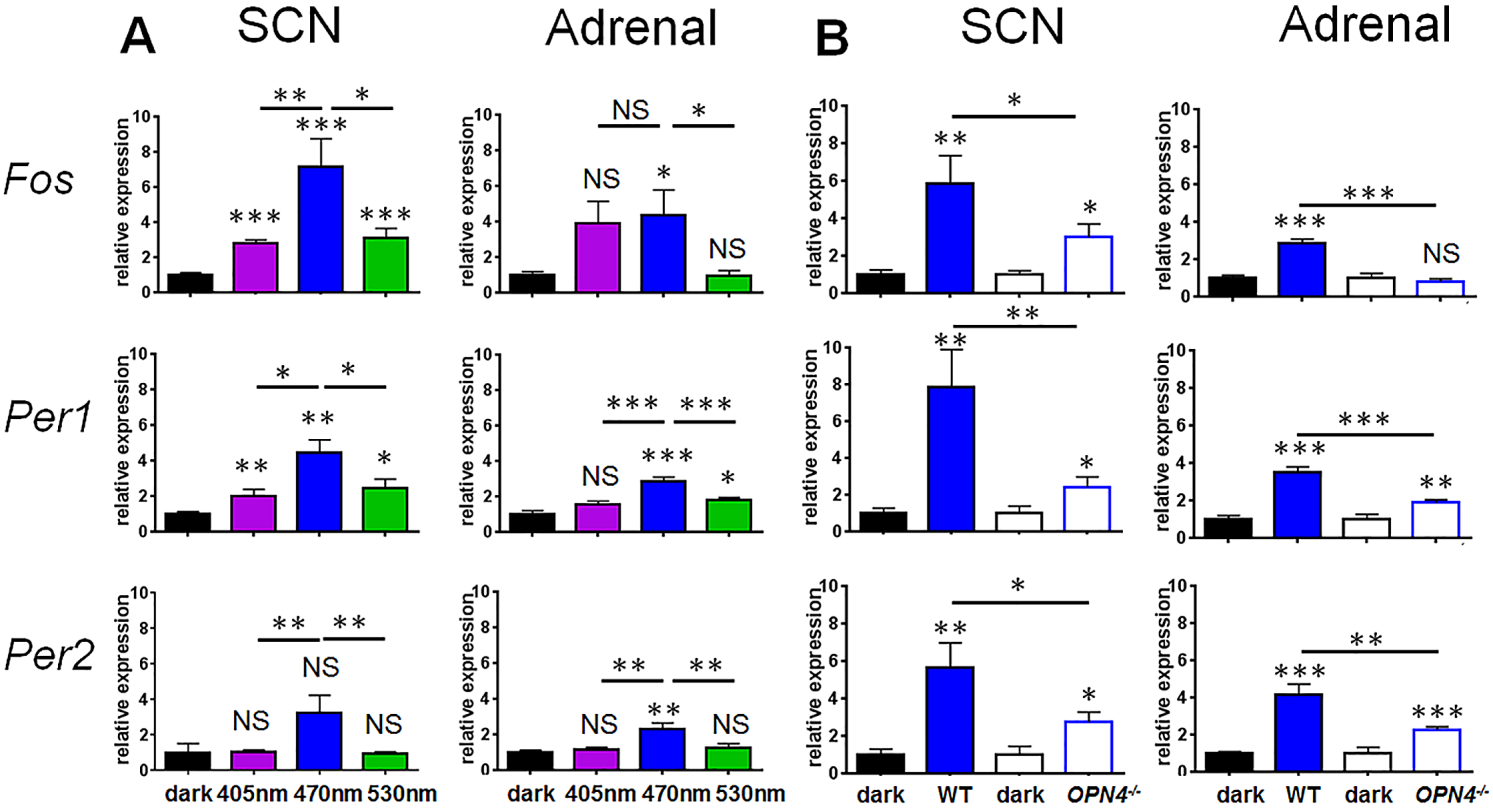
Wavelength-dependent effects on immediate early gene expression in the SCN and adrenal gland. (**A**) Relative mRNA expression of *Fos*, *Per1*, and *Per2* after 1 hr monochromatic light pulse in the SCN and adrenal gland. One-way ANOVA for wavelength, *Fos* (F_(3.18)_ = 23.615 *p* ≤ 0.001), posthoc Tukey dark versus violet *p* ≤ 0.001, dark versus blue *p* ≤ 0.001, dark versus green *p* ≤ 0.001; *Per1* (F_(3.23)_ = 10.366, *p* ≤ 0.001) dark versus violet *p* = 0.036, dark versus blue *p* ≤ 0.001, dark versus green *p* = 0.007); *Per2* (F_(3.19)_ = 5.715 *p* = 0.006) dark versus violet *p* = 0.196, dark versus blue *p* ≤ 0.001, dark versus green *p* = 0.265). A significant effect of wavelength was observed in the SCN for all three genes (one-way ANOVA for wavelength, *Fos* F_(2,14)_ = 8.603, *p* = 0.004; *Per1* F_(2,19)_ = 4.740, *p* = 0.021; *Per2* F_(2,14)_ = 8.565, *p* = 0.004), with blue light showing significantly greater *Fos*, *Per1*, and *Per2* expression in the SCN compared to either violet (posthoc Tukey *Fos p* = 0.006, *Per1 p* = 0.014, *Per2 p* = 0.008) or green light (posthoc Tukey *Fos p* = 0.012, *Per1 p* = 0.028, *Per2 p* = 0.008). In the adrenal gland, responses to blue light were significantly greater than either green or violet light. Both blue and green light induced *Per1* expression (one-way ANOVA for wavelength, *Per1* F_(3,36)_ = 13.070, *p* < 0.001; posthoc Tukey dark versus violet *p* = 0.098, dark versus blue *p* < 0.001, dark versus green *p* = 0.023). Blue light produced significantly greater *Per1* induction compared to either green or violet light (green versus blue *p* = 0.001, violet versus blue *p* < 0.001). *Fos* and *Per2* expression were also induced by blue light but not violet or green light (one way ANOVA for wavelength *Fos* F_(3.31)_ = 2.516 *p* = 0.076, dark versus blue *p* = 0.045; *Per2* F_(3.31)_ = 7.033 *p* = 0.001, dark versus blue *p* = 0.004). Neither violet nor green light produced a significant induction of *Fos* or *Per2* expression (*Fos* dark versus violet *p* = 0.063, dark versus green *p* = 0.979, *Per2* dark versus violet *p* = 0.961, dark versus green *p* = 0.998). (**B**) Responses to blue light were subsequently studied in *Opn4*^*-/-*^ mice (open bars) compared with wildtype mice (solid bars). *Opn4*^*-/-*^ mice showed light induced gene expression for all three genes in the SCN, but responses were significantly attenuated in comparison to wildtype animals (unpaired *t* test wildtype versus *Opn4*^*-/-*^ *Fos p* = 0.029, *Per1 p* = 0.007, *Per2 p* = 0.029). In the adrenal gland *Per1* and *Per2* (but not *Fos*) were induced in response to light. All three genes showed attenuated responses in comparison to wildtype mice (unpaired *t* test wildtype versus *Opn4*^*-/-*^ *Fos p* < 0.001, *Per1 p* < 0.001, *Per2 p* = 0.002). The geometric mean of three housekeeping genes *Actb*, *Gapdh*, and *Arbp* were used for normalisation. Statistically significant changes in gene expression versus control (dark) conditions are shown by symbols directly above histogram bars, whereas comparisons between wavelengths are indicated by horizontal lines. Data plotted as mean ± SEM. *N* = 5–12/group. Significant differences indicated by *** *p* ≤ 0.001, ** *p* ≤ 0.01, * *p* ≤ 0.05, NS = not significant. The data used to make this figure can be found in S4 Data.

To determine if the different wavelengths of light result in different effects at a molecular level, we measured the expression of the immediate early gene *Fos*, and the light-regulated clock genes *Per1* and *Per2* following a 1 h light pulse in the SCN and adrenal gland (Fig 4A). Consistent with previous studies, all three wavelengths evoked increased expression of *Fos*, *Per1*, and *Per2* in the SCN. Different wavelengths resulted in differential levels of induction of all three genes in the SCN, with blue light evoking significantly greater increases compared to either violet or green light. Violet and green light resulted in comparable levels of gene induction in the SCN.

Nocturnal light exposure has previously been shown to result in induction of immediate early genes in the adrenal gland [19,25]. We reasoned that if light input is relayed from the retina to the adrenal gland via the SCN, light-induced changes in the adrenal should reflect those observed in the SCN. In the adrenal gland, we found that responses to blue light were significantly greater than to either green or violet light. Both blue and green light induced *Per1* expression, with significantly greater *Per1* induction compared to either green or violet light. *Fos* and *Per2* expression were also induced by blue light, but not violet or green light.

### Gene Induction in Response to Blue Light Is Melanopsin-Dependent

Given the high-amplitude changes in gene expression in response to blue light, we next investigated whether these responses were melanopsin-dependent. We compared gene expression in the SCN and adrenal gland between wildtype and *Opn4*^*-/-*^ mice exposed to blue light (Fig 4B). As the previously observed molecular responses to violet and green light were of much lower amplitude, these were not studied, as it would be extremely difficult to attain sufficient statistical power to resolve any difference. In response to blue light, *Opn4*^*-/-*^ mice showed light-induced gene expression for all three genes in the SCN. However, these responses were attenuated compared to wildtype animals. In the adrenal gland, only *Per1* and *Per2* were increased in comparison to dark controls in response to light. All three genes showed attenuated responses in comparison to wildtype mice. Together, these results show that molecular responses to blue light are impaired in *Opn4*^*-/-*^ mice at the level of both SCN and adrenal gland. The effects of light on SCN and adrenal, and the high levels of gene induction in response to blue light in particular, suggested that the behavioural responses to blue light may be accompanied by changes in plasma corticosterone.

### The Effects of Light on Plasma Corticosterone Are Wavelength-Dependent

**Fig 5:**
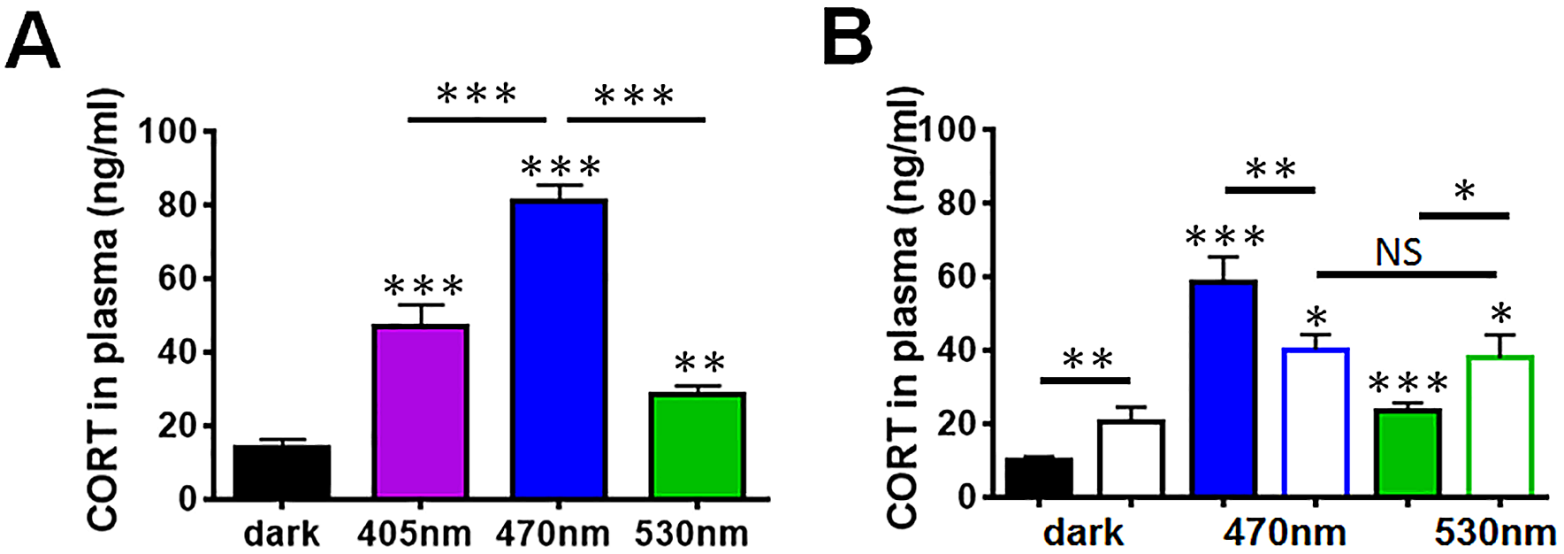
Wavelength-dependent effects on plasma corticosterone. (**A**) Plasma corticosterone levels were assessed from terminal blood following a 30 min light pulse at ZT14. All three wavelengths produced significant rise in plasma corticosterone levels. However, blue light evoked significantly higher corticosterone secretion compared to violet, green, and dark conditions (one-way ANOVA for wavelength, F_(3.30)_ = 39.80, *p* ≤ 0.001. Posthoc Tukey dark versus violet *p* ≤ 0.001, dark versus blue *p* ≤ 0.001, dark versus green *p* = 0.002, violet versus blue *p* ≤ 0.001, and blue versus green *p* ≤ 0.001). Responses to green light were significantly lower compared to either violet or blue light. (**B**) *Opn4*^*-/-*^ mice (open bars) show not only an overall elevation of plasma corticosterone levels in response to light (two-way ANOVA for wavelength and genotype, wavelength effect F_(2.36)_ = 42.135 *p* ≤ 0.001, posthoc Tukey *Opn4*^*-/-*^ dark versus blue *p* = 0.001, *Opn4*^*-/-*^ dark versus green *p* = 0.013) but also showed different responses to wildtype mice (open bars, wavelength x genotype interaction F_(2.36)_ = 13.809 *p* ≤ 0.001). Responses to blue light were significantly attenuated (wildtype versus *Opn4*^*-/-*^ blue *p* = 0.004) whereas responses to green light were elevated (*p* = 0.029). As such, there was no significant difference in corticosterone response between blue and green light in *Opn4*^*-/-*^ mice. Baseline levels of corticosterone at ZT14 were significantly elevated in *Opn4*^*-/-*^ mice compared to wildtype controls (*p* = 0.017). Histograms reflect mean ± SEM, *n* = 5–10/group. Significant differences indicated by *** *p* ≤ 0.001, ** *p* ≤ 0.01, * *p* ≤ 0.05, NS = not significant. The data used to make this figure can be found in S5 Data.

Previous studies have shown that acute white light exposure at ZT16 produces an increase in plasma corticosterone levels related to light-induced adrenal *Per1* expression [19,20,25]. To determine if the observed sleep, behavioural, and molecular responses to blue light are accompanied by a rise in plasma corticosterone, we exposed wildtype C57BL/6 mice to 1 h of violet, blue or green light. All three wavelengths produced significantly elevated plasma corticosterone levels compared with dark-exposed controls (Fig 5A). However, blue light resulted in significantly higher corticosterone levels compared to violet, green or dark conditions. Responses to green light were significantly lower compared to either violet or blue light.

### Melanopsin Mediates the Effects of Light on Plasma Corticosterone

To investigate whether the effects of different wavelengths of light on *Opn4*^*-/-*^ mice are reflected at the level of plasma corticosterone, we measured plasma corticosterone levels in *Opn4*^*-/-*^ mice exposed to either blue or green light (Fig 5B). *Opn4*^*-/-*^ mice still show an elevation of plasma corticosterone levels in response to light, but responded differently in comparison to wildtype mice. Responses to blue light were significantly attenuated in *Opn4*^*-/-*^ animals, although responses to green light were enhanced. As a result of these changes, no significant differences in corticosterone levels between blue and green stimuli were apparent in *Opn4*^*-/-*^ mice. This finding is consistent with the loss of wavelength-dependent effects of light on sleep induction and behavioural light aversion in melanopsin-deficient animals. Surprisingly, baseline levels of corticosterone at ZT14 were slightly elevated in *Opn4*^*-/-*^ mice compared to wildtype controls.

### Different Pathways Mediate the Wake-Promoting Versus Sleep-Promoting Effects of Light

**Fig 6:**
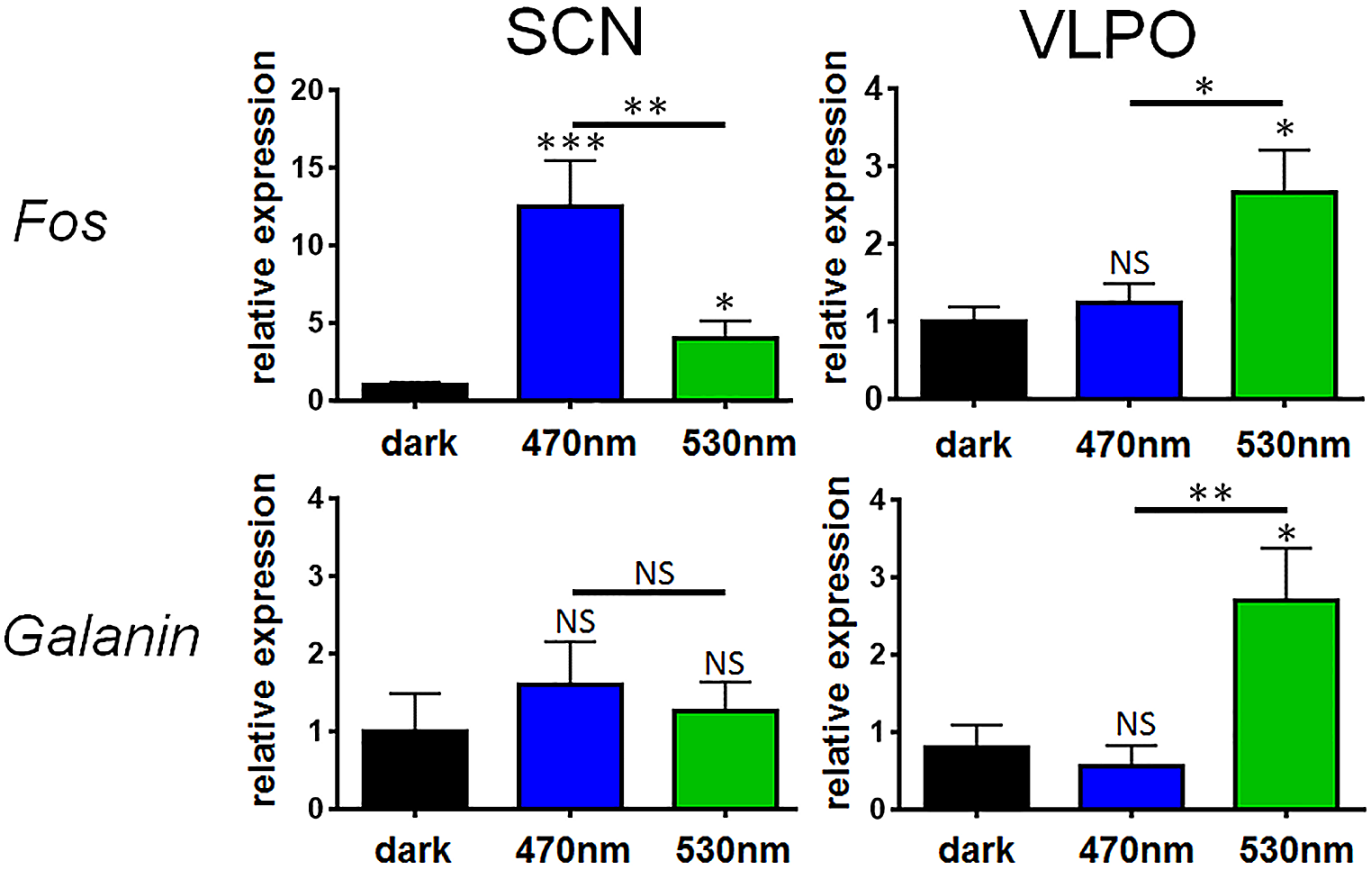
Molecular responses to light in SCN and VLPO are wavelength-dependent. A *Fos* induction in response to a 30 min light pulse was studied in wildtype mice exposed to blue or green light in both the SCN and VLPO (two-way ANOVA for wavelength x brain region interaction F_(2,31)_ = 10.968 *p* ≤ 0.001). Mice exposed to blue light show greater *Fos* induction in the SCN compared with green light (posthoc Tukey dark versus blue *p* = 0.001, dark versus green *p* = 0.035, blue versus green *p* = 0.008). By contrast, in the VLPO, a greater response to green light than blue light was observed (dark versus blue *p* = 0.880, dark versus green *p* = 0.015). As a control, *Gal* induction was also studied, showing no induction in the SCN but significant induction in the VLPO (two-way ANOVA for wavelength and x brain region interaction F_(2,30)_ = 3.774 *p* = 0.035). This response was only evident in response to green light (posthoc Tukey dark versus blue *p* = 0.742, dark versus green *p* = 0.011, blue versus green *p* = 0.003). Histograms show mean ± SEM, *n* = 5–8/group. Significant differences indicated by *** *p* ≤ 0.001, ** *p* ≤0.01, * *p* ≤0.05, NS = not significant. The data used to make this figure can be found in S6 Data.

To determine if blue and green light exert different effects at the level of retinorecipient targets, we studied responses to these wavelengths at the level of the SCN and VLPO (Fig 6). Mice were exposed to blue and green light stimuli and after 30 min SCN and VLPO were collected for gene expression analysis using quantitative PCR (qPCR). Consistent with our previous findings (Fig 4A), we found that blue light produced a larger *Fos* induction in the SCN than green light. However, in the VLPO we found a greater response to green light than blue light. As the sleep active neurons of the VLPO are galaninergic [26], we also studied expression of *Gal* in both SCN and VLPO. In the VLPO (but not the SCN), we found that *Fos* induction was accompanied by increased *Gal* expression. These data suggest that the different behavioural effects of blue and green light are mirrored by molecular responses at the level of the SCN and VLPO, suggesting that different pathways may mediate the wake-promoting and sleep-promoting effects of light on behaviour.

### Blocking the Action of Glucocorticoids Reduces Light-Induced Sleep Latency

It is unclear whether the inhibitory effects of blue light on sleep are mediated via elevated plasma corticosterone or whether elevated corticosterone levels are a downstream consequence of the heightened state of arousal produced by this stimulus. To investigate this question, we blocked the effects of corticosterone via systemic injection of the glucocorticoid receptor antagonist mifepristone (RU-486) in wildtype C57BL/6 mice at ZT12. These mice were then exposed to a blue light pulse at ZT14. In comparison to vehicle-treated animals, mifepristone-treated mice showed elevated basal sleep (S5 Fig). However, they did show significantly enhanced sleep onset in comparison to vehicle treated animals (two-way ANOVA for treatment and time, treatment F_(1.98)_ = 16.473, *p* ≤ 0.001, posthoc Tukey drug versus control first 10 min pulse *p* = 0.003, 10–20 min *p* ≤ 0.001, 20–30 min pulse *p* = 0.01). These findings show that the effects of blue light on sleep induction can be attenuated by blocking the effects of elevated plasma corticosterone.

## Discussion

Here, we show that sleep induction and light aversion responses in mice are differentially affected by wavelength. Green light is associated with rapid sleep induction, whereas blue light results in an arousal response, characterised by reduced sleep, behavioural light aversion, and elevated plasma corticosterone. Notably we show that in melanopsin-deficient animals, these opposing effects of wavelength are abolished. As such, melanopsin-deficient animals show attenuated sleep induction under green light or enhanced sleep onset under blue light. These data suggest that the effects of nocturnal light exposure are more complex than first envisaged, and that sleep induction in rodents depends upon a balance between these arousal-promoting and sleep-promoting effects of light.

Given the established role of melanopsin in the regulation of sleep in response to light [4–6], we predicted that blue light would be most effective at inducing sleep in mice. To our surprise, we found that blue light was less effective, with a delayed sleep onset compared to other wavelengths. These findings appear to be due to a specific arousal-promoting response to blue light, which inhibits the normal sleep-promoting effects of light. In support of this hypothesis, the delayed sleep induction in response to blue light exposure is accompanied by behavioural light aversion, increased immediate early gene expression in both the SCN and adrenal gland, and elevated levels of plasma corticosterone. By contrast, green light results in rapid sleep induction in the home cage or transient increases in exploratory behaviour in a novel environment. These responses are accompanied by markedly lower immediate early gene induction in the SCN and adrenal gland and low levels of plasma corticosterone.

The arousing effects of blue light suggest a potential role for melanopsin and, as predicted, we found that these responses were attenuated in melanopsin-deficient mice. As this arousal response to blue light normally inhibits sleep induction, melanopsin-deficient mice show enhanced sleep induction. Whilst this may appear to contradict previously published findings [4–6], this is not the case. We show that melanopsin-deficient mice do indeed show attenuated sleep induction under green or white light. This can be explained by the fact that most white light sources have an emission spectrum optimised for human photopic vision (with a peak sensitivity ~555 nm), which will be more comparable to our 530 nm rather than 470 nm wavelength stimulus [15]. To test this assumption, we compared the light available to each photoreceptor class from our different wavelength stimuli with that from commonly used white light sources—incandescent, fluorescent, and LED. As predicted, these white light sources are more comparable with the green (530 nm) stimulus used in this study than the blue (470 nm) (S6A and S6B Fig). As such, sleep induction in melanopsin-deficient mice appears dependent upon the spectral power distribution of the light source used and may depend upon the balance of sleep-promoting and arousal-promoting effects of different wavelengths. In addition, previous studies have shown that melanopsin-deficient mice show normal behavioural light aversion under white light conditions [27]. This is consistent with the data presented here, where over the whole timecourse of light exposure under green light, melanopsin-deficient mice show light aversion comparable to wildtype animals.

There is considerable evidence that the SCN relays photic information via the autonomic nervous system (ANS) to the adrenal gland to regulate corticosterone secretion in response to light [19,25,28]. This pathway is comparable to the well-known pathway mediating melatonin suppression in response to light, involving signals relayed via the SCN and sympathetic nervous system [2]. In agreement with these findings, we show that gene expression in both the SCN and adrenal gland is significantly increased after light exposure, and that the effects of different wavelengths of light on behaviour are mirrored by molecular responses in SCN and adrenal as well as in plasma corticosterone. In addition, we show that elevated responses to blue light are attenuated in melanopsin-deficient mice. This is the first demonstration that melanopsin plays a role in the regulation of adrenal corticosterone in response to light, adding to the repertoire of physiological and behavioural responses involving these photoreceptors. Glucocorticoids are important mediators of homeostasis and stress. They exert their complex effects via glucocorticoid receptors, which are expressed in both neurons and glia in the brain [29]. To determine if the increase in corticosterone observed is a downstream marker of the arousing effects of blue light, or whether corticosterone exerts a direct effect on the regulation of sleep, we assessed responses to blue light following administration of a glucocorticoid receptor antagonist. This resulted in enhanced sleep induction in response to blue light, suggesting that glucocorticoids exert a direct effect on the regulation of sleep. These changes in sleep latency appear to fall within the timescale of transcriptional regulation in response to glucocorticoid signalling. Moreover, recent data on the effects of stress on sleep have shown that chronic corticosterone administration affects sleep via activation of the locus coeruleus (LC), which in turn suppresses GABAergic neurons of the VLPO. In this study, RU486 was shown to inhibit the effects of corticosterone on sleep by blocking the action of glucocorticoid receptors at the level of the LC. This provides a putative mechanism by which corticosterone induction in response to light may inhibit sleep induction [30].

Our data suggest that melanopsin provides an important drive for arousal in response to light. In response to blue light, arousal is high, and in the absence of melanopsin, this arousal response is attenuated. By contrast, the attenuated sleep responses to green and white light seen in melanopsin-deficient mice are more difficult to account for and do not appear to be due to impaired melanopsin signalling to the VLPO as previously assumed. Due to the overlapping spectral sensitivity of rods, cones, and melanopsin, high irradiance stimuli of different wavelengths will be expected to exert comparable effects on different non–image-forming responses. The finding that different wavelengths of light exert opposing effects on these responses could be due to either spectral opponency at the level of the retina or SCN, or independent pathways involving different photoreceptor projections. Whilst spectral opponency is consistent with recent findings of colour-sensitive neurons within the SCN [31], it seems unlikely that this can account for our findings, as increased responses to shorter wavelength violet light would be expected, and these were not observed in the present study. By contrast, different non–image-forming responses to light are thought to be mediated via different retinorecipient areas of the brain, with the SCN mediating circadian entrainment and corticosterone induction and the VLPO mediating sleep responses [7,28,32]. This provides a plausible mechanism by which melanopsin projections to the SCN may mediate the arousing effects of light (including the elevation of plasma corticosterone), whereas rod–cone signals may mediate acute sleep induction via activation of the VLPO. This mechanism is supported by the differing *Fos*-induction responses observed between the SCN and VLPO. Given the known projections of melanopsin pRGCs to the VLPO [32], such responses could arise from different pRGC subtypes to the SCN and VLPO, with SCN input from M1 pRGCs and VLPO input from non-M1 cells, which may be more dependent upon rod/cone input [33]. Moreover, whilst attention has focused on the sparse innervation of the VLPO by melanopsin pRGCs, information on the innervation of the VLPO by non-melanopsin RGCs is limited [26]. Finally, it is also possible that photic information from the retina may mediate VLPO responses indirectly via its wide range of inputs [34].

If melanopsin mediates the arousal-promoting effects of blue light, why do melanopsin-deficient mice show attenuated responses to green light? Unlike the innervation of the SCN by M1 pRGCs mediating the arousing responses to blue light, the pRGC subtype innervating the VLPO may be more dependent on rod and cone input in addition to its intrinsic photosensitivity. Melanopsin has recently been shown to provide an independent measure of irradiance to provide optimal settings for visual circuits [18]. If rod and cone signals are primarily mediating the sleep-promoting effects of light, a loss of melanopsin-dependent light adaptation may account for the impaired sleep induction observed in this and previous studies on the role of melanopsin in sleep. Under these bright light conditions, rods and cones will be saturated as they will be unable to adapt. This explanation is attractive as it also accounts for the loss of chromatic responses observed in melanopsin-deficient mice. This finding suggests that under bright light conditions, specific deficits may occur in wavelength discrimination in the absence of melanopsin. Finally, it should not be overlooked that the elevated basal corticosterone levels in melanopsin-deficient mice may also inhibit the sleep-promoting effects of green light.

**Fig 7:**
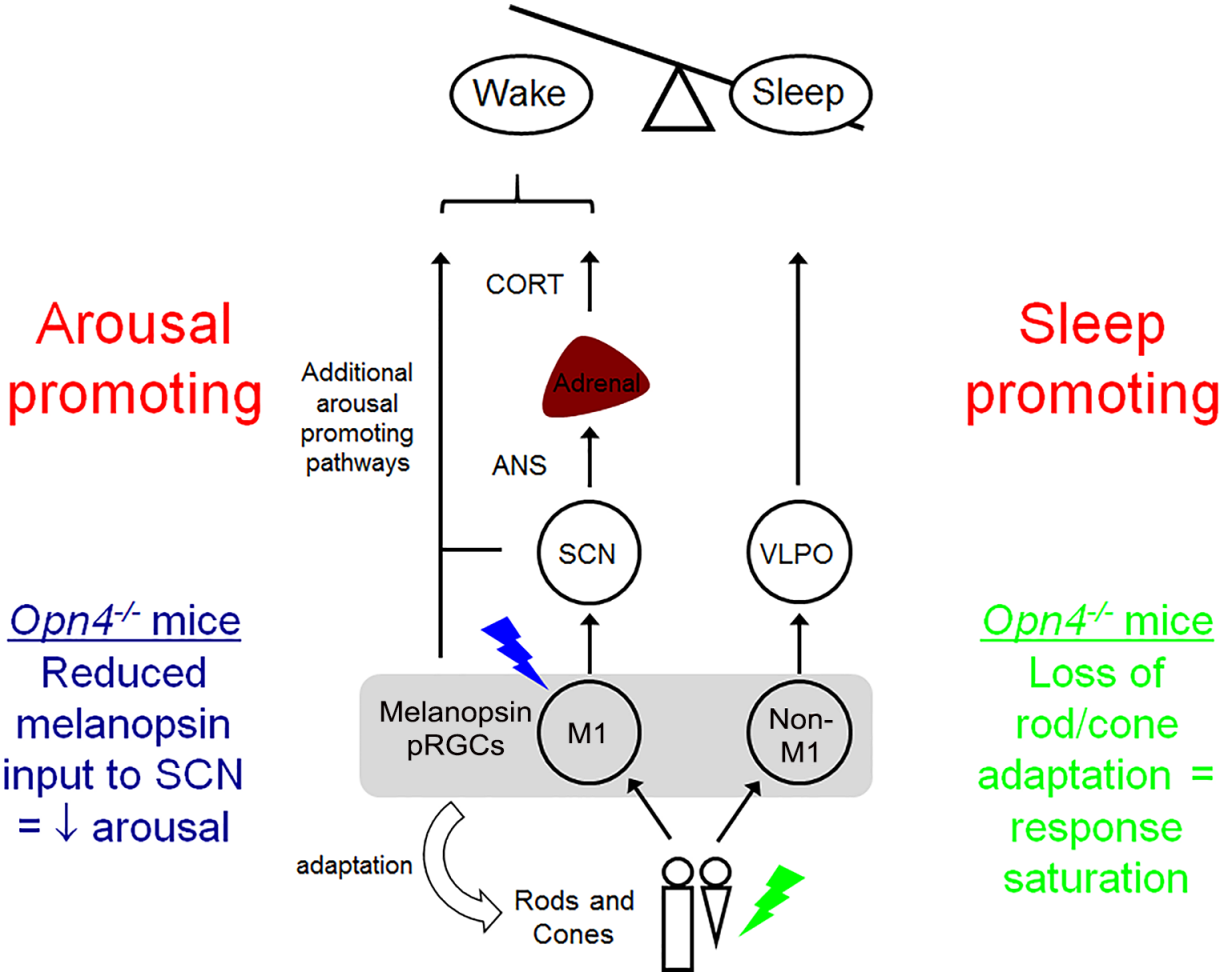
The arousal-promoting and sleep-promoting effects of nocturnal light exposure in mice depend upon different neural pathways. In response to blue light, M1 pRGC projections to the SCN result in activation of the adrenal gland via the ANS. This alertness promoting pathway is associated with corticosterone release and increased waking. This pathway is strongly activated by blue light due to the spectral sensitivity of melanopsin which peaks ~480 nm. Loss of melanopsin results in reduced activation of this pathway, resulting in enhanced sleep induction. Additional arousal-promoting pathways undoubtedly contribute to this response. By contrast, green light results in activation of the sleep-promoting VLPO pathway, most likely via non-M1 melanopsin pRGCs that are more dependent upon rod and cone input. As melanopsin plays a critical role in rod and cone adaptation, under bright light conditions, loss of melanopsin results in attenuated sleep induction via this pathway. Moreover, the resultant saturation of rod and cone pathways also results in a loss of chromatic responses.

Our findings can be summarised as a model incorporating both arousal-promoting and sleep-promoting pathways (Fig 7). This model accounts for the opposing effects of different wavelengths of light, whereby blue light enhances arousal via SCN projecting M1 pRGCs, resulting in elevated corticosterone via autonomic innervation of the adrenal. Clearly, we would also anticipate that additional arousal pathways may be involved. For example, melanopsin pRGCs also innervate the subparaventricular zone (SPZ), which has a direct projection to the dorsomedial hypothalamus (DMH) that in turn regulates both VLPO and lateral hypothalamus (LH) [35]. By contrast, we propose that green light promotes sleep induction via non-M1 pRGCs that are more dependent upon rod/cone input, which in turn project to the VLPO. These data are supported by the differential effects of blue and green light on *Fos* induction in the SCN and VLPO, respectively. In melanopsin-deficient animals, the arousal-promoting response to blue light is attenuated, resulting in an enhanced sleep onset (due to a reduced arousal response). Moreover, melanopsin is also critical for adaptation of rod/cone pathways, and under bright light conditions, the absence of melanopsin results in attenuated sleep induction to green light. This in turn also accounts for the loss of chromatic responses observed in melanopsin-deficient mice. This model provides a tentative explanation for our data. However, given that different pRGC subtypes show an overlap in their projections [36], a simple model based upon mutually antagonistic responses is perhaps overly simplistic, particularly given that the subtypes of pRGC projecting to the VLPO remain largely uncharacterised. Behavioural light aversion (mediated via projections to regions such as the amygdala) and adrenal corticosterone responses (mediated via the SCN and sympathetic nervous system) may involve separate neuronal circuits operating over different timescales. However, there is considerable evidence of an intimate relationship between adrenal stress hormones and arousal pathways involving the amygdala [37,38]. As such, we propose that these pathways operate to provide a consistent behavioural response to promote arousal to environmental stimuli, but given the diversity of pathways known to modulate sleep and arousal [35], the model we propose in Fig 7 is likely to be an oversimplification.

It is unclear why separate neural pathways are necessary to regulate arousal- and sleep-promoting pathways. One possible explanation is that the detection of environmental irradiance required for non–image-forming responses such as circadian entrainment and hormonal responses are well-served by the response characteristics of the melanopsin pRGC system. However, sleep involves reciprocal interactions between a wide range of sleep- and wake-promoting brain regions, giving rise to a “flip-flop” switch [39]. This bistable feedback loop is thought to be important in preventing intermediate states, minimising the time during which transition states between sleep and waking states occur. As such, the acute switch between sleep states in response to light is very different from other non–image-forming responses, as the critical signal is the transition between dark and light rather than ambient irradiance levels. As rods and cones will provide more reliable indicators of light transitions, this may account for their greater role in sleep induction. Consistent with this explanation, melanopsin is essential for the maintenance of light-induced sleep, where long-term environmental irradiance is a more reliable stimulus [40].

It is also possible to place our results into a broader ecological context. Around twilight, there is a relative enrichment of blue light across the dome of the sky [41]. This would result in a change in the balance between blue light- and green light-sensitive pathways (S6C Fig). The spectral sensitivity of the blue light-aversive pathways may act to delay mice emerging from their burrows during early dusk and encourage a retreat to their burrow at early dawn. Such responses may help reduce the risk of predation at dawn and dusk, when there is a conflicting requirement for exploratory activity and safety. By contrast, exposure to longer wavelengths (green or white light) during the day may serve to limit daytime activity during waking bouts and promote sleep.

Human studies have demonstrated an important role of light in the regulation of arousal. This involves activation of multiple brain areas involved in alertness, cognitive function, and melatonin production [42–47]. These studies report an increased effect of short wavelength light (470 nm or lower) associated with the increased suppression of melatonin, reduction in subjective sleepiness, reduced reaction times, and changes in EEG power in the delta-theta frequency range [43,44,48]. Moreover, blue light exposure prior to sleep has been shown to reduce slow wave activity in the 0.75–5.5 Hz range in the first sleep cycle with a subsequent increase in the third sleep cycle. REM sleep duration during these cycles was also observed to be shortened [49]. These findings are consistent with recent studies on the effects of light-emitting devices on both melatonin rhythms and sleep latency [50]. These data suggest a key role of blue light exposure in inducing arousal and wakefulness in humans and are supported by our findings in mice. As C57BL/6 mice are naturally melatonin deficient [51], the mechanisms mediating this effect of blue light are unlikely to be melatonin dependent. However, our data suggest that despite the differences between nocturnal and diurnal species, light may play a similar alerting role in mice as has been shown in humans, providing a valuable animal model to study the effects of light on cognitive function [48]. Whilst the arousal-promoting effects of light are consistent with human data, the sleep-promoting effects of light may differ between nocturnal and diurnal species. Here, we show that despite the arousal-promoting effects of blue light, mice still eventually go to sleep in response to nocturnal light exposure. By contrast, in diurnal species, nocturnal light may be expected to promote wakefulness, which may therefore be potentiated rather than antagonised by the arousing effects of blue light.

In summary, here we show that light may have either arousal-promoting or sleep-promoting effects on mice, with corresponding changes in behaviour and molecular responses, including the regulation of adrenal corticosterone. These opposing effects of light are dependent upon wavelength, with blue and green light resulting in arousal and sleep, respectively. These coordinated responses appear to involve different neural pathways, which are both impaired in melanopsin-deficient mice. This results in a reduction in the arousal-promoting effects of blue light, as well as attenuated sleep-promoting responses to green light. In addition to providing the first demonstration of a role for melanopsin in the regulation of adrenal corticosterone, these findings challenge our existing understanding of the role of melanopsin in the regulation of sleep. Finally, the identification of a light-dependent arousal-promoting mechanism in mice provides a valuable animal model to study the effects of light on human cognitive brain function. With the increasing demands of the 24/7 society resulting in rising levels of both artificial lighting and shift work, there is an urgent need to understand the biological effects of artificial light on sleep versus alertness.

## Materials and Methods

### Animals

All experiments were conducted in accordance with the Home Office (UK) regulations, under the Animals (Scientific Procedures) Act of 1986. All procedures were reviewed by the clinical medicine animal care and ethical review body (AWERB), and were conducted under PPL 30/2812, under PILs IF656800A and I869292DB. Wild type male C57BL/6 mice (3/7 mo old) and melanopsin knockout (*Opn4*^*-/-*^) on a C57BL/6 background (ten generations of backcrossing) were used throughout. Before all experiments, mice were singly housed under a 12:12 light dark cycle with white light 250 lux when measured at the bottom of the cage, with food and water available ad libitum. Animals were housed in specific pathogen free (SPF) conditions, and the only reported positives on health screening over the entire time course of these studies were for *Helicobacter hepaticus* and *Entamoeba sp* (Envigo, Alconbury UK).

### Experimental Setup for Sleep Assessment

To assess behavioural responses (including activity, sleep induction/duration and light aversion) to different wavelengths of light we monitored home cage behaviour with miniature near infrared (NIR) video cameras (Sentient Mini night vision CCTV camera, Maplin, UK) mounted above each cage. Behaviour was tracked for 3 h and analysed in real time using ANY-maze (Version 4.98, Stoelting, US). Sleep onset was determined from episodes of immobility of 40 s or longer, and data were grouped into 10 min bins. This method has previously been validated, showing that the extended immobility correlates very highly (correlation coefficient = 0.95) with EEG/EMG under both baseline conditions as well as following administration of pharmacological agents. Moreover, we have also shown that this method is capable of accurately identifying the dose-dependent effects of sedatives, stimulants and light [52].

### Acute Light Pulse Studies and Sleep Onset Determination

To estimate the effect of monochromatic light pulses on immobility induction we housed *Opn4*^*-/-*^ and WT mice under a LD cycle (white light 250 lux). During video tracking, all mice have been kept in polypropylene cages, and the top of the cage was open so that the entire cage could be used as open field arena. The water bottle as well as the feeder (a drawer) were installed outside of the cage. White light as well as monochromatic light (530 nm, 470 nm, and 405 nm) were provided using cool white LEDs (Luxeon Star, Quadica Developments Inc, Brantford, Ontario). These wavelengths were used to produce spectral separation between stimuli, due to the overlapping photopigment absorption curves. The photon flux of all three monochromatic LEDs was isoquantal at 14.9 log quanta when measured at the bottom in the middle of the cage. The wavelength as well as the photon flux were measured using a calibrated Ocean Optics spectrometer. For acclimatisation in the new cages mice were kept for 5 days under a white light LD cycle (~250 lux). A 1 h monochromatic light pulse was administered at ZT14. Mice which exhibited behaviourally-defined sleep prior to the light pulse were excluded from the analysis, as light was found to result in waking (rather than sleep) under such conditions. As such, unlike circadian responses to light, the behavioural state of animals prior to light exposure was found to be critical when considering sleep induction. Therefore, only mice which showed activity 10 min before light pulse were considered for sleep analysis. Moreover, as baseline sleep is lowest at the start of the dark phase, sleep induction light pulses were administered at ZT14 (rather than later in the dark phase) to coincide with this period of greatest waking. Sleep latency for each animal was determined as the time during which the first bout of behaviourally-defined sleep occurred. Total sleep was defined as the total period of behaviourally-defined sleep during the 1 h light pulse.

### Behavioural Light Aversion

Light aversion was measured in naive mice using a dark chamber light chamber paradigm as previously reported [53]. Behavioural testing was performed for 10 min at ZT14 and analysed using ANYmaze software. To estimate the difference in aversion behaviour induced by monochromatic light, the testing was performed with 470 nm, 405 nm, and 530 nm. Control studies were performed with no light. Each mouse was gently placed into the illuminated chamber of the box facing the portal to the dark chamber. A video tracking system (see experimental setup for behavioural test) was used to monitor behaviour and to quantify the total time that mice spent in the illuminated versus the dark chamber during a 10 min trial. The chambers were thoroughly cleaned between animals.

### Tissue and Blood Collection

For ex vivo phase analysis we exposed wildtype and *Opn4*^*-/-*^ to a 1 h light pulse and collected trunk blood after 30–40 min, the time of peak corticosterone induction [19,25]. Dark-treated mice were used as a control. A parallel set of mice was equally treated with light pulse, and one hour after light exposure (the expected peak of *Fos* and *Per1*) SCN and adrenal gland were collected and used for *Per1*, *Per2*, and *Fos* gene expression analysis using qPCR.

### SCN Collection

After 1 hr light pulse, animals were killed under dim light by cervical dislocation. To prevent any photic stimulation to the SCN, eyes were immediately removed. Removed brains were placed into a brain matrix (Kent Scientific, Torrington CT, US). Two skin graft blades (Kent Scientific, Torrington CT, US) one positioned at Bregma −0.10 mm and the second at Bregma −1.10 caudal from the first, were used to cut a 1 mm thick brain slice. SCN punches were collected from the brain slices by using a sample corer (1 mm internal diameter, Fine Science Tools GmbH, Heidelberg, Germany). SCN punches were stored at −80°C prior to RNA extraction.

### VLPO Collection

Wildtype mice were exposed to a 1hr green light pulse before SCN and VLPO punches were collected 30–40 min after the end of the light pulse. This time point is characterised by a peak of *Fos* in the SCN [5,54,55]. To prevent any photic stimulation to the SCN eyes were immediately removed. Three skin graft blades were used (Kent Scientific, Torrington CT, USA) one positioned at Bregma −0.10mm and the second at Bregma −1.10 caudal from the first for the SCN (as described above), and the third 1 mm before 1.10 mm for VLPO. Two 1 mm thick brain slices were then dissected. SCN as well VLPO punches were collected as described previously [5,55] using a sample corer (1mm internal diameter, Fine Science Tools GmbH, Heidelberg, Germany). Samples were stored at −80°C prior RNA extraction.

### RNA Extraction and Sample Preparation

Total RNA of SCN and VLPO punches was extracted using the microRNeasy column method (QIAGEN, Hilden, Germany). Total RNA of adrenal gland was extracted using the miniRNeasy column method (QIAGEN, Hilden, Germany). The quantity of RNA was estimated using Nanodrop1000 (Thermo Fisher Scientific, Waltham, MA USA).

### Quantitative PCR

cDNA was synthesised from RNA samples with a qScript cDNA synthesis kit (Quanta Biosciences, Gaithersburg, MD). qPCR was performed with Sybr green and SDS7700 thermal cycler (Applied Biosystems, Foster City, CA). Relative quantification of transcript levels was conducted as described previously [56]. The geometric mean of three housekeeping genes *Actb*, *Gapdh*, and *Arbp* was used for normalisation. *Gal*a*nin* was used as a positive control as described in previous studies [6]. Expression levels were normalised to control (dark) values. *Per1*, *Per2*, *Fos* and *Gal* expression were estimated using following primers: *Per1* forward AGTTCCTGACCAAGCCTCGTTAG, Per1 reverse CCTGCCCTCTGCTTGTCATC, *Per2* forward GGGGTGAGATTCGTCATTGAACTTG, *Per2* reverse AGGACATTGGCACACTGGAAAGAG, *Fos* forward ATCGGCAGAAGGGGAAAGTAG, *Fos* reverse GCAACGCAGACTTCTCATCTTCAAG, *Gal* forward ATGCCTGCAAAGGAGAAGAGAGGT, *Gal* reverse TCTGTGGTTGTCAATGGCATGTGG.

### Measurement of Plasma Corticosterone

Trunk blood was collected in microfuge tube containing anticoagulant EDTA (10 ul for 500 ul blood), kept for 10 min on ice and centrifuged. Obtained plasma was stored at −20°C until corticosterone measurement. Corticosterone was measured from 1:10 diluted samples using an ELISA kit (Assaypro LLC St. Charles, MO) according to manufacturer’s instructions.

### RU-486 Application

To block glucocorticoid receptor mediated effects, we injected mice with the glucocorticoid antagonist RU-486 (Sigma Aldrich) at ZT12 and started measuring behaviour from ZT13 until ZT15. RU-486 was administered as described previously [57]. Briefly, RU-486 was dissolved in 20% dimethyl sulfoxide (DMSO)/80% polythelyne glucol (PEG)-300. Drug dosage (100 mg/kg body weight) was based on the previous studies [57,58]. As control the same amount of (1 ml/kg body weight) of vehicle (saline diluted in 20%DMSO/80%(PEG)-300) was injected.

### Calculation of Effects of Polychromatic Light Sources

To determine the light available to the different photoreceptors of the mouse retina for the different wavelength stimuli used in this study as well as white light sources, the Rodent Toolbox v1.0 was used [15]. For the 405 nm, 470 nm, and 530nm LEDs used in this study, spectral power distributions were measured using a calibrated Ocean Optics spectrometer. For white light sources, standard illuminant functions were used from the Toolbox. For daylight and twilight data, previously published data were used [59]. Comparable results for daylight were obtained using the D65 standard illuminant function. Data were expressed in alpha-opic lux. However, as it is not possible to directly compare different photoreceptor channels, these were expressed as relative to the total alpha-opic lux across all four channels.

### Statistical Analysis

Results are presented as mean ± SEM. Statistical analysis was performed using SPSS Statistics 22.0. All data for statistical analysis were first checked for normal distribution using Kolmogorov Smirnov test. Non-normally distributed data were log transformed prior to statistical analysis to ensure normality and homogeneity of variance. Statistical significance of group comparisons was tested with one-way ANOVA and posthoc Tukey. Multivariate experiments such as genotype and treatment were analysed using two-way ANOVA with Tukey’s posthoc analysis. Time course experiments such as across multiple hours or min were analysed using rep. ANOVA. Assessment of relative photoreceptor activity under the 405 nm, 470 nm, and 530 nm wavelengths has been performed using the previously published rodent toolbox [15].

## Supporting Information

S1 Data Excel spreadsheet containing, in separate sheets, data for Fig 1A–1C and underlying raw values used to generate averages for sleep profiles, sleep latency, and sleep duration in WT mice. (XLSX)

S2 Data Excel spreadsheet containing, in separate sheets, data for Fig 2A–2E and underlying raw values to generate averages for sleep profiles, sleep latency, and sleep duration in WT- and melanopsin-deficient mice. (XLSX)

S3 Data Excel spreadsheet containing, in separate sheets, data for Fig 3B–3E and underlying raw values to generate averages for time spent in the hidden zone in the 1st minute and in the following 9 min. (XLSX)

S4 Data Excel spreadsheet containing, in separate sheets, relative *Fos*, *Per1*, and *Per2* gene expression; data for Fig 4A–4B. (XLSX)

S5 Data Excel spreadsheet containing, in separate sheets, data for Fig 5A–5B and underlying raw values of optical density (OD) and corticosterone concentration to generate averages for corticosterone concentration. (XLSX)

S6 Data Excel spreadsheet containing relative *Fos* and *Galanin* gene expression in the SCN and the VLPO; data for Fig 6. (XLSX)

S7 Data Excel spreadsheet containing, in separate sheets, data to generate S1A Fig (spectral power distribution of LED stimuli used) and S1B Fig (relative photoreceptor activity produced by 405 nm, 470 nm, and 530 nm). (XLSX)

S8 Data Excel spreadsheet containing, in separate sheets, data for S2A–S2C Fig and underlying raw values to generate averages for sleep profiles, sleep latency and sleep duration under blue and green light exposure at ZT22. (XLSX)

S9 Data Excel spreadsheet containing, in separate sheets, data for S3A–S3F Fig and underlying raw values to generate averages for white light effect on sleep profiles, sleep latency, and sleep duration in WT- and melanopsin-deficient mice as well as for violet, blue, and green light effect on sleep profiles, sleep latency, and sleep duration in melanopsin-deficient mice. (XLSX)

S10 Data Excel spreadsheet containing data for latency to first entry during behavioural light aversion in WT mice S4 Fig. (XLSX)

S11 Data Excel spreadsheet containing, in separate sheets, data for S5 Fig and underlying raw values to generate averages for sleep profiles affected by RU-486 and vehicle treatment under blue light exposure. (XLSX)

S12 Data Excel spreadsheet containing, in separate sheets, data for relative alpha opic lux using white light sources incandescent S6A Fig, fluorescent and cool-white LED S6B Fig as well as daylight versus twilight data S6C Fig. (XLSX)

S1 Fig Different wavelength stimuli used in this study. (**A**) Spectral power distribution of LED stimuli used. Three different wavelengths were used: 405 nm (violet), 470 nm (blue), and 530 nm (green). All stimuli were confirmed using a calibrated spectrometer. (**B**) Relative photoreceptor activity produced by 405 nm (violet), 470 nm, (blue), and 530 nm (green) with similar photon flux of ~14.9 log quanta. The data used to make this figure can be found in S7 Data.
(TIF)

S2 Fig Effects of different wavelength light on sleep at ZT22. (**A**) Mice exposed to blue (470 nm) light for 1 hr at ZT22 showed delayed sleep onset compared to green (530 nm) light. (**B**) Comparable to ZT14, sleep induction was delayed in response to blue light compared with green light. (**C**) Total sleep duration during the 1 h light pulse was unchanged under both lightning conditions. Data plotted as mean percentage ± SEM (*n* = 8–9/group). Horizontal black-white-black bar illustrates the light pulse condition from ZT22 until ZT23; blue symbols and histograms represent 470 nm lighting condition, green symbols, and histograms represent 530 nm lighting condition. Data analysed using unpaired *t* test, **p ≤ 0.01, NS = not significant. The data used to make this figure can be found in S8 Data.
(TIF)

S3 Fig Effect of white light on sleep induction in *Opn4*^*-/-*^ mice. (**A**) Sleep onset latency induced by acute white light exposure in WT and *Opn4*^*-/-*^ mice at ZT14. *Opn4*^*-/-*^ deficient as well as WT mice were exposed to 1 hr white light (~250 lux) at ZT14. (**B**) *Opn4*^*-/-*^ mice showed delayed sleep onset under white light exposure compared to wildtype mice (**A**,**C**), which resulted in reduction of sleep duration in *Opn4*^*-/-*^ mice. (*n* = 8/group) (open circles illustrate *Opn4*^*-/-*^, solid circles illustrate WT mice, symbols represent mean ± SEM, horizontal black-white-black bar represents light pulse duration from ZT14 until ZT15, *p ≤ 0.05, unpaired *t* test). (**D**,**E**,**F**) Sleep profile during acute monochromatic light exposure in *Opn4*^*-/-*^ mice at ZT14. *OPN4*^*-/-*^ mice were exposed to three different wavelengths: violet, green, and blue at ZT14. All three different wavelengths evoked comparable sleep profile, sleep onset and duration in *Opn4*^*-/-*^ (*n* = 8/group). Horizontal black-white-black bar represents light pulse duration from ZT14 until ZT15, symbols represent mean ± SEM, One-way ANOVA, posthoc Tukey, NS = not significant. The data used to make this figure can be found in S9 Data.
(TIF)

S4 Fig Latency to first entry during behavioural light aversion. The latency from placing the mouse into the box until the first entry to the hidden zone was significantly shorter under blue illumination compared to violet, green light and control = dark condition. One-way ANOVA for light condition, F_(3.25)_ = 20.70, *p* ≤ 0.001. Posthoc Tukey blue versus control *p* = 0.021, violet versus blue *p* ≤ 0.001, blue versus green *p* ≤ 0.001. Histograms reflect mean percentage ± SEM of latency to first entry in the dark box of the light dark box during the 10 min trial (*n* = 6–10/group). Two-way repeated measures ANOVA, posthoc Tukey. ***p ≤ 0.001, *p ≤ 0.05. The data used to make this figure can be found in S10 Data.
(TIF)

S5 Fig Prolonged sleep latency in response to blue light is dependent upon glucocorticoid receptor activation. RU-486 effect on sleep induction during blue light pulse. To investigate the reinforcing effect of blue light-induced plasma corticosterone, the glucocorticoid receptor antagonist RU-486 (100 mg/kg body weight, solid circles) and/or vehicle (control group, open squares) were administrated to C57BL/6 WT at ZT12. At ZT14 both animal groups were exposed to 1 hr acute blue light pulse. Mice treated with RU-486 exhibited reduced activity compared to control group. During the light pulse, sleep induction was significantly enhanced in RU-486 treated mice compared to the control group. Data plotted as mean ± SEM, horizontal blue bar illustrates the blue light pulse duration from ZT14 until ZT15 *n* = 8/group. The data used to make this figure can be found in S11 Data.
(TIF)

S6 Fig Light available to different retinal photoreceptors under different lighting conditions. (**A**) Different wavelength stimuli used in this study. (**B**) Commonly used white light sources incandescent, fluorescent, and cool-white LED, which are designed to be enriched with longer wavelengths for human vision. (**C**) Daylight versus twilight data, showing the effects of the relative enrichment of blue light. Data are expressed in alpha-opic lux, normalised to 100%, as described in the Materials and Methods. The data used to make this figure can be found in S12 Data.
(TIF)

S1 Video Video demonstrates behavioural response to acute blue (470 nm) light exposure in WT mouse. The original video of 3 h have been cut down to 1 h and 4 min (2 min darkness before and after 1 h light pulse) and accelerated 8 times. The acute light exposure is indicated by text in the upper left corner in the video. After acute blue light exposure, WT mouse continues showing activity that lasts for 17 min and is followed by sleep induction. The general activity lasted for 14–15 min before sleep induction. Noticeable shortly before sleep induction WT mouse exhibits intensive grooming.
(MP4)

S2 Video Video demonstrates behavioural response to acute green (530 nm) light exposure in WT mouse. The original video of 3 h have been cut down to 1 h and 4 min (2 min darkness before and after 1 h light pulse) and accelerated eight times. The acute light exposure is indicated by text in the upper left corner in the video. WT mouse exposed to green light shows a reduction of activity due to light exposure that is transformed to a rapid sleep induction after 2.5 min. The general activity lasted for 14–15 min before sleep induction. Noticeable shortly before sleep induction WT mouse exhibits intensive grooming.
(MP4)

S3 Video Video demonstrates behavioural response to acute blue (470 nm) light exposure in *OPN4*^*-/-*^ mouse. The original video of 3 h have been cut down to 1 h and 4 min (2 min darkness before and after 1 h light pulse) and accelerated eight times. The acute light exposure is indicated by text in the upper left corner in the video. *Opn4*^*-/-*^ mouse exposed to blue light exhibits comparable change of activity to behaviourally defined-sleep compared to *Opn4*^*-/-*^ mouse exposed to green light (S4 Video). The general activity lasted for 14–15 min before sleep induction. Noticeable shortly before sleep induction *Opn4*^*-/-*^ mouse exposed exhibits intensive grooming.
(MP4)

S4 Video Video demonstrates behavioural response to acute green (530 nm) light exposure in *OPN4*^*-/-*^ mouse. The original video of 3 h have been cut down to 1 h and 4 min (2 min darkness before and after 1 h light pulse) and accelerated eight times. The acute light exposure is indicated by text in the upper left corner in the video. *Opn4*^*-/-*^ mouse exposed to green light exhibits comparable change of activity to behaviourally defined-sleep compared to *Opn4*^*-/-*^ mouse exposed to blue light (S3 Video). The general activity lasted for 14–15 min before sleep induction. Noticeable shortly before sleep induction *Opn4*^*-/-*^ mouse exposed green light, respectively, exhibit intensive grooming.
(MP4)

## Abbreviations

ANS: autonomic nervous system
DMH: dorsomedial hypothalamus
LC: locus coeruleus
LH: lateral hypothalamus
NIR: near infrared
OD: optical density
pRGC: photosensitive retinal ganglion cell
qPCR: quantitative PCR
SCN: suprachiasmatic nuclei
SPF: specific pathogen free
SPZ: subparaventricular zone
VLPO: ventrolateral preoptic area
