# Supplementary figures and images for: Melanopsin Regulates Both Sleep-Promoting and Arousal-Promoting Responses to Light
Source: PLoS Biol. 2016 Jun 8;14(6):e1002482. doi: 10.1371/journal.pbio.1002482 (PMC4898879; doi:10.1371/journal.pbio.1002482)

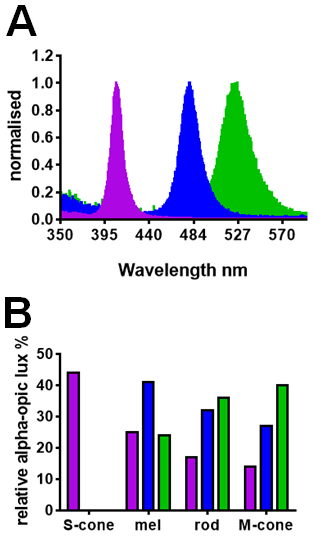

Supplement: S1 Fig — (A) Spectral power distribution of LED stimuli used. Three different wavelengths were used: 405 nm (violet), 470 nm (blue), and 530 nm (green). All stimuli were confirmed using a calibrated spectrometer. (B) Relative photoreceptor activity produced by 405 nm (violet), 470 nm, (blue), and 530 nm (green) with similar photon flux of ~14.9 log quanta. The data used to make this figure can be found in S7 Data. (TIF) [file pbio.1002482.s013.tif]

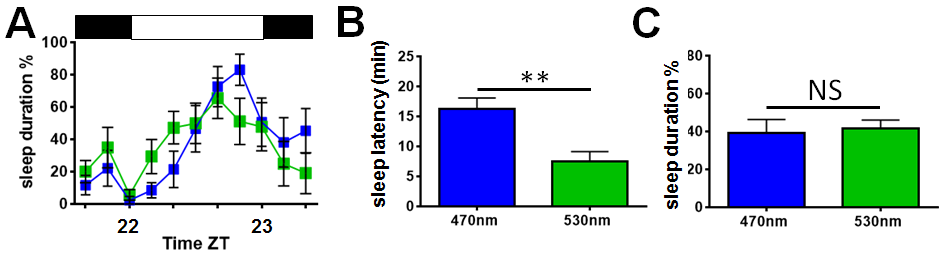

Supplement: S2 Fig — (A) Mice exposed to blue (470 nm) light for 1 hr at ZT22 showed delayed sleep onset compared to green (530 nm) light. (B) Comparable to ZT14, sleep induction was delayed in response to blue light compared with green light. (C) Total sleep duration during the 1 h light pulse was unchanged under both lightning conditions. Data plotted as mean percentage ± SEM (n = 8–9/group). Horizontal black-white-black bar illustrates the light pulse condition from ZT22 until ZT23; blue symbols and histograms represent 470 nm lighting condition, green symbols, and histograms represent 530 nm lighting condition. Data analysed using unpaired t test, **p ≤ 0.01, NS = not significant. The data used to make this figure can be found in S8 Data. (TIF) [file pbio.1002482.s014.tif]

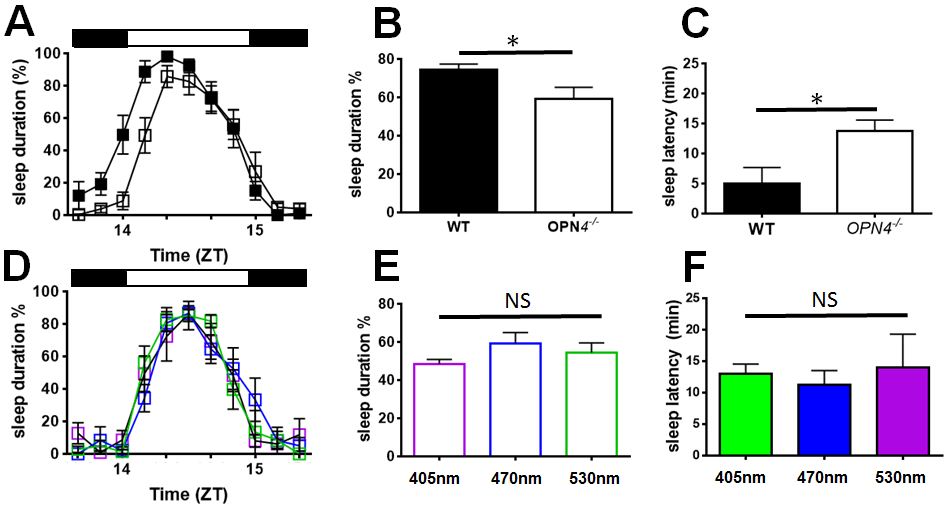

Supplement: S3 Fig — (A) Sleep onset latency induced by acute white light exposure in WT and Opn4-/- mice at ZT14. Opn4-/- deficient as well as WT mice were exposed to 1 hr white light (~250 lux) at ZT14. (B) Opn4-/- mice showed delayed sleep onset under white light exposure compared to wildtype mice (A,C), which resulted in reduction of sleep duration in Opn4-/- mice. (n = 8/group) (open circles illustrate Opn4-/-, solid circles illustrate WT mice, symbols represent mean ± SEM, horizontal black-white-black bar represents light pulse duration from ZT14 until ZT15, *p ≤ 0.05, unpaired t test). (D,E,F) Sleep profile during acute monochromatic light exposure in Opn4-/- mice at ZT14. OPN4-/- mice were exposed to three different wavelengths: violet, green, and blue at ZT14. All three different wavelengths evoked comparable sleep profile, sleep onset and duration in Opn4-/- (n = 8/group). Horizontal black-white-black bar represents light pulse duration from ZT14 until ZT15, symbols represent mean ± SEM, One-way ANOVA, posthoc Tukey, NS = not significant. The data used to make this figure can be found in S9 Data. (TIF) [file pbio.1002482.s015.tif]

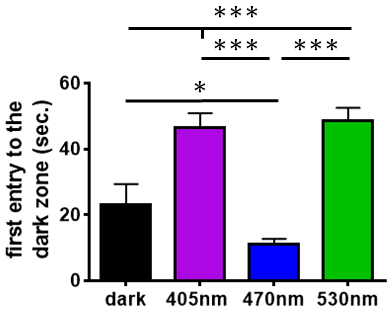

Supplement: S4 Fig — The latency from placing the mouse into the box until the first entry to the hidden zone was significantly shorter under blue illumination compared to violet, green light and control = dark condition. One-way ANOVA for light condition, F(3.25) = 20.70, p ≤ 0.001. Posthoc Tukey blue versus control p = 0.021, violet versus blue p ≤ 0.001, blue versus green p ≤ 0.001. Histograms reflect mean percentage ± SEM of latency to first entry in the dark box of the light dark box during the 10 min trial (n = 6–10/group). Two-way repeated measures ANOVA, posthoc Tukey. ***p ≤ 0.001, *p ≤ 0.05. The data used to make this figure can be found in S10 Data. (TIF) [file pbio.1002482.s016.tif]

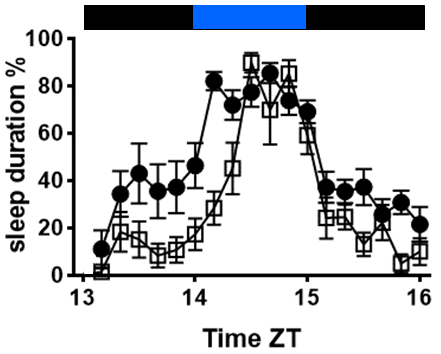

Supplement: S5 Fig — RU-486 effect on sleep induction during blue light pulse. To investigate the reinforcing effect of blue light-induced plasma corticosterone, the glucocorticoid receptor antagonist RU-486 (100 mg/kg body weight, solid circles) and/or vehicle (control group, open squares) were administrated to C57BL/6 WT at ZT12. At ZT14 both animal groups were exposed to 1 hr acute blue light pulse. Mice treated with RU-486 exhibited reduced activity compared to control group. During the light pulse, sleep induction was significantly enhanced in RU-486 treated mice compared to the control group. Data plotted as mean ± SEM, horizontal blue bar illustrates the blue light pulse duration from ZT14 until ZT15 n = 8/group. The data used to make this figure can be found in S11 Data. (TIF) [file pbio.1002482.s017.tif]

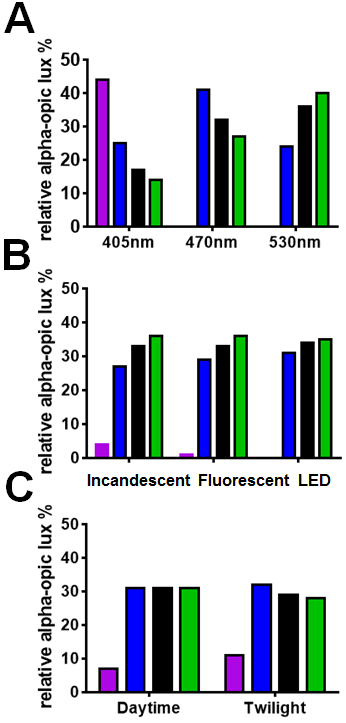

Supplement: S6 Fig — (A) Different wavelength stimuli used in this study. (B) Commonly used white light sources incandescent, fluorescent, and cool-white LED, which are designed to be enriched with longer wavelengths for human vision. (C) Daylight versus twilight data, showing the effects of the relative enrichment of blue light. Data are expressed in alpha-opic lux, normalised to 100%, as described in the Materials and Methods. The data used to make this figure can be found in S12 Data. (TIF) [file pbio.1002482.s018.tif]
